# The clinicopathological and prognostic value of the pretreatment neutrophil-to-lymphocyte ratio in small cell lung cancer: A meta-analysis

**DOI:** 10.1371/journal.pone.0230979

**Published:** 2020-04-02

**Authors:** Yan Lu, JinWen Jiang, ChaoXiang Ren

**Affiliations:** Clinical Laboratory, DongYang People’s Hospital, Dongyang, Zhejiang, China; Chang Gung Memorial Hospital at Linkou, TAIWAN

## Abstract

Although many scholars have recently studied the relationships between the pretreatment neutrophil-to-lymphocyte ratio (NLR) and prognosis in patients with small cell lung cancer (SCLC), the conclusions have been inconsistent. Accordingly, in this meta-analysis, we attempted to assess the clinicopathological and prognostic value of the pretreatment NLR in SCLC. Related literature was searched using PubMed, Embase, Cochrane Library, Web of Science, Chinese Biomedical Literature, China National Knowledge Infrastructure (CNKI), and Wanfang databases. Each eligible study was extracted, and a meta-analysis was performed using hazard ratios (HRs) and 95% confidence intervals (95% CIs) to assess the prognostic value of NLR. Evaluation of the clinicopathological significance of NLR in SCLC used odds ratios (ORs) and 95% confidence intervals (95% CIs). We included a total of 20 studies with 21 outcomes (5141 patients) in this meta-analysis. The results showed that high pretreatment NLR was closely related to poorer progression free survival (PFS) and overall survival (OS) (PFS, HR = 1.55, 95% CI = 1.27–1.88, *P* < 0.0001; I^2^ = 0%; OS, HR = 1.40, 95% CI = 1.26–1.55, *P* < 0.00001; I^2^ = 64%). In addition, pretreatment NLR was significantly associated with clinical stage of SCLC (OR = 2.14, 95% CI = 1.35–3.39, *P* = 0.001). Our meta-analysis showed that high levels of pretreatment NLR were significantly associated with a more serious clinical stage and poorer PFS and OS in SCLC.

## Introduction

Small cell lung cancer (SCLC) is a highly malignant neuroendocrine tumor, accounting for 15–20% of lung cancer cases [1]. Compared with non-small cell lung cancer, SCLC exhibits more aggressive invasiveness, earlier distant metastasis, and poorer prognosis. Despite the continuous development of medical technology, the prognosis of SCLC is still not optimistic, and the median overall survival is often less than 6 months[2]. Thus, the identification of novel biomarkers for predicting prognosis is essential for improving long-term outcomes. Although some new biomarkers have been shown to be independent prognostic factors for SCLC[3, 4], most of these biomarkers are expensive and time-consuming to detect. Therefore, identification of inexpensive and simple biomarkers for SCLC may have important clinical implications.

In recent years, the association between systemic inflammation and tumors has become an essential research hotspot. Many studies have shown that inflammation is involved in all aspects of tumor development[5, 6]. The neutrophil-to-lymphocyte ratio (NLR) is a simple, widely available clinical indicator of inflammation and has been shown to be associated with the prognosis of a variety of malignant tumors[7–9]. Recently, many scholars have evaluated the relationships between pretreatment NLR and prognosis in patients with SCLC; however, the conclusions have been inconsistent.

Therefore, we conducted this meta-analysis in order to assess the clinicopathological and prognostic value of the pretreatment NLR in SCLC.

## Materials and methods

### Search strategies

We searched the PubMed, Embase, Cochrane Library, Web of Science, Chinese Biomedical Literature, China National Knowledge Infrastructure (CNKI), and Wanfang databases. The search was performed using a combination of the following technical terms: (“Neutrophil-lymphocyte ratio” OR NLR OR “Neutrophil lymphocyte ratio” OR “Neutrophil to lymphocyte ratio”) and (“Lung Cancer” OR “Lung Carcinoma” OR “Small Cell Lung Cancer” OR “Oat Cell Lung Cancer” OR “Small Cell Cancer Of The Lung” OR “Carcinoma, Small Cell Lung” OR “Oat Cell Carcinoma of Lung” OR "Small Cell Lung Carcinoma"). The search time limit was from the time of establishment of the database to February 1, 2020. There was no geographical restriction on the literature search; however, only Chinese and English studies were considered. Additionally, the references included in the literature were retrieved to avoid missing detection.

### Inclusion and exclusion criteria

The inclusion criteria were as follows: (1) the subject was a patient who had been diagnosed with SCLC; (2) the pretreatment NLR value was obtained; (3) the purpose of the study was to explore the relationships between NLR and OS or PFS in SCLC; (4) the hazard ratio (HR) and 95% confidence interval (95% CI) were reported in the literature; and (5) the language of the document was English or Chinese.

The exclusion criteria were as follows: (1) case reports, letters, reviews, meta-analyses, and conference reports; (2) duplicate publications; and (3) unable to obtain the full text or data from the text.

### Data extraction and quality assessment

Two authors independently used the Newcastle-Ottawa Scale (NOS) [10] to assess the quality of the studies. If the authors disagreed, the disagreement was resolved through discussion. The following data were extracted: first author, publication year, country, ethnicity, age, sample size, follow-up (months), median OS (months), cut-off value, clinical stage (limited stage or/and extensive stage)[11], type of survival analysis, and outcome. The study was considered to be of high quality when the NOS score was greater than or equal to 6.

### Statistical analysis

The HR and 95% CI were pooled to assess the prognostic value of NLR for patients with SCLC. Evaluation of the clinicopathological significance of NLR in SCLC used odds ratios (ORs) and 95% confidence intervals (95% CIs). We used Cochran’s Q statistic test and then analyzed the heterogeneity between studies based on I^2^ and *P* values [12]. According to the Cochrane Handbook [13], when I^2^ was less than or equal to 50% and the *P*-value was greater than 0.10, the heterogeneity was acceptable. When the different studies included were not heterogeneous, they were combined using a fixed-effects model; otherwise, random-effects models were used [14], and subgroup analyses [15] and meta-regression [16] were used to discuss heterogeneity sources. When the combined HR was greater than 1, the survival rate was poor. If the 95% CI did not contain 1, the result was considered statistically significant. Sensitivity analysis was also needed to assess whether the results were stable. At the same time, quantitative analysis of publication bias was performed using Begg’s tests [17] and Egger’s tests [18], and if necessary, the trim and fill method [19] was used to quantitatively analyze publication bias. Results with *P* values of less than 0.05 were considered statistically significant. Stata 12.0 statistical analysis software (Stata Corporation, College Station, TX, USA) and Review Manager software (version 5.3; Cochrane Collaboration, London, UK) were used for all studies.

## Results

### Study search

**Fig 1:**
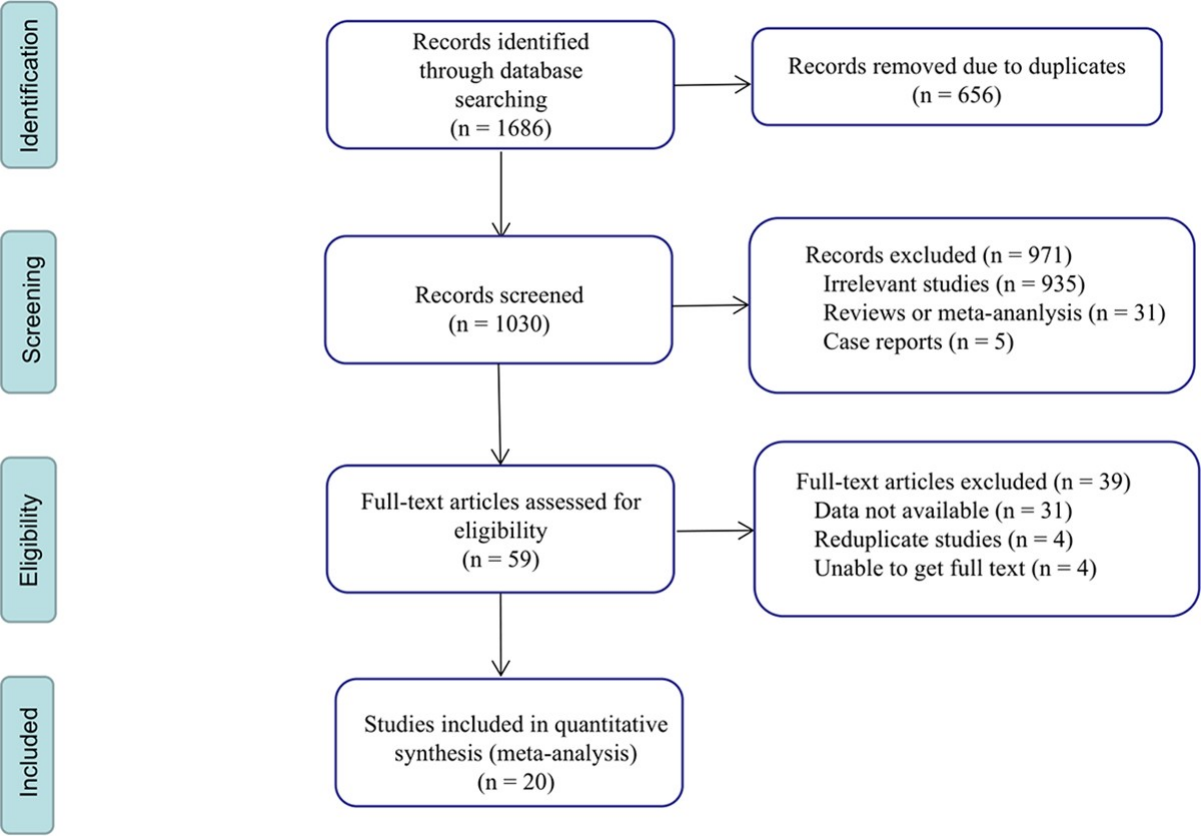
The flow chart of the study selection process.

In total, 1686 studies were retrieved according to the search strategy. According to the inclusion and exclusion criteria, after reviewing duplicates and screening the titles or abstracts, 59 research articles were evaluated. Finally, 20 eligible studies were included [20–39], with 21 outcomes. The flow chart for the study is shown in Fig 1.

### Study characteristics

This meta-analysis included 5141 patients with SCLC (3461 men and 1680 women). The number of patients in each study ranged from 52 to 919, with a median of 172. The cut off value of NLRs ranged from 2.258 to 5.0, with a median of 3.70. The main characteristics of the incorporated literature are shown in Table 1.

**Table 1:** Characteristics of all included studies in the meta-analysis.

First Author	Year	Country	Ethnicity	Age (range and median)	Sample Size	Follow-up (months)	Median OS (months)	Cut off	Clinical stage	Survival analysis	Outcome	NOS
Ju[20]	2018	China	Asian	NR	154	NR	NR	3.70	L+E	M	OS	8
Wang (1)[21]	2017	China	Asian	31–83	172	NR	19.18	3.86	L+E	M	OS	7
Huang[22]	2016	China	Asian	31–88	112	NR	NR	4.50	L+E	M	OS	8
Zhang[23]	2017	China	Asian	59 (30–78)	265	NR	16	4.0	L+E	M	OS	8
Wang (2)[24]	2016	China	Asian	62 (28–79)	153	NR	23.3	3.20	L+E	M	OS/PFS	8
Wang (3)[25]	2017	China	Asian	NR	181	NR	NR	3.60	L+E	M	OS	8
Bernhardt[26]	2018	Germany	Caucasian	64 (37–93)	350	NR	20	4.0	L	U	OS	7
Murray[27]	2014	UK	Caucasian	61.6 (38.3–77.4)	52	26.1	21.1	5	L	M	OS	6
Hong[28]	2015	China	Asian	56 (16–84)	919	NR	10.4	5	L+E	M	OS	8
Sakin[29]	2019	Turkey	Caucasian	61 (35–83)	113	6 (1–33)	NR	3	E	M	OS	8
Suzuki {1}[30]	2018	USA	Caucasian	63	252	NR	11.0	4.0	E	M	OS	8
Wang (4)[31]	2014	China	Asian	NR	114	NR	14	3	L+E	M	OS	8
Xie ①[32]	2015	China	Asian	68 (27–91)	555	10.8	NR	5	E	M	OS	8
Xie ②[32]	2015	China	Asian	68 (27–91)	383	10.8	NR	5	L	M	OS	8
Suzuki {2}[33]	2018	USA	Caucasian	65	122	NR	16.6	2.9	L	M	OS	7
Käsmann[34]	2017	Germany	Caucasian	NR	65	NR	20	4.0	L	M	OS	8
Deng[35]	2017	China	Asian	58 (24–81)	320	39.1	13.8	2.65	L+E	M	OS/PFS	8
Lohinai[36]	2019	Hungary	Caucasian	58	155	NR	NR	2.258	L+E	M	OS	7
Wang (5)[37]	2019	China	Asian	58(39–71)	228	46	20	2.3	L+E	M	OS/PFS	8
Li[38]	2019	China	Asian	NR	160	NR	NR	2.32	L+E	M	OS	7
Liu[39]	2019	China	Asian	59	316	NR	11	2.68	L+E	M	OS	8

(1)–(5): different authors and different studies; {1}–{2}: the same author but different studies; ①–②: the same author and the same study; L: limited stage; E: extensive stage; M: multivariate; U: univariate; NR: not reported; NOS: Newcastle-Ottawa scale

### The prognostic value of the pretreatment NLR in SCLC

**Fig 2:**
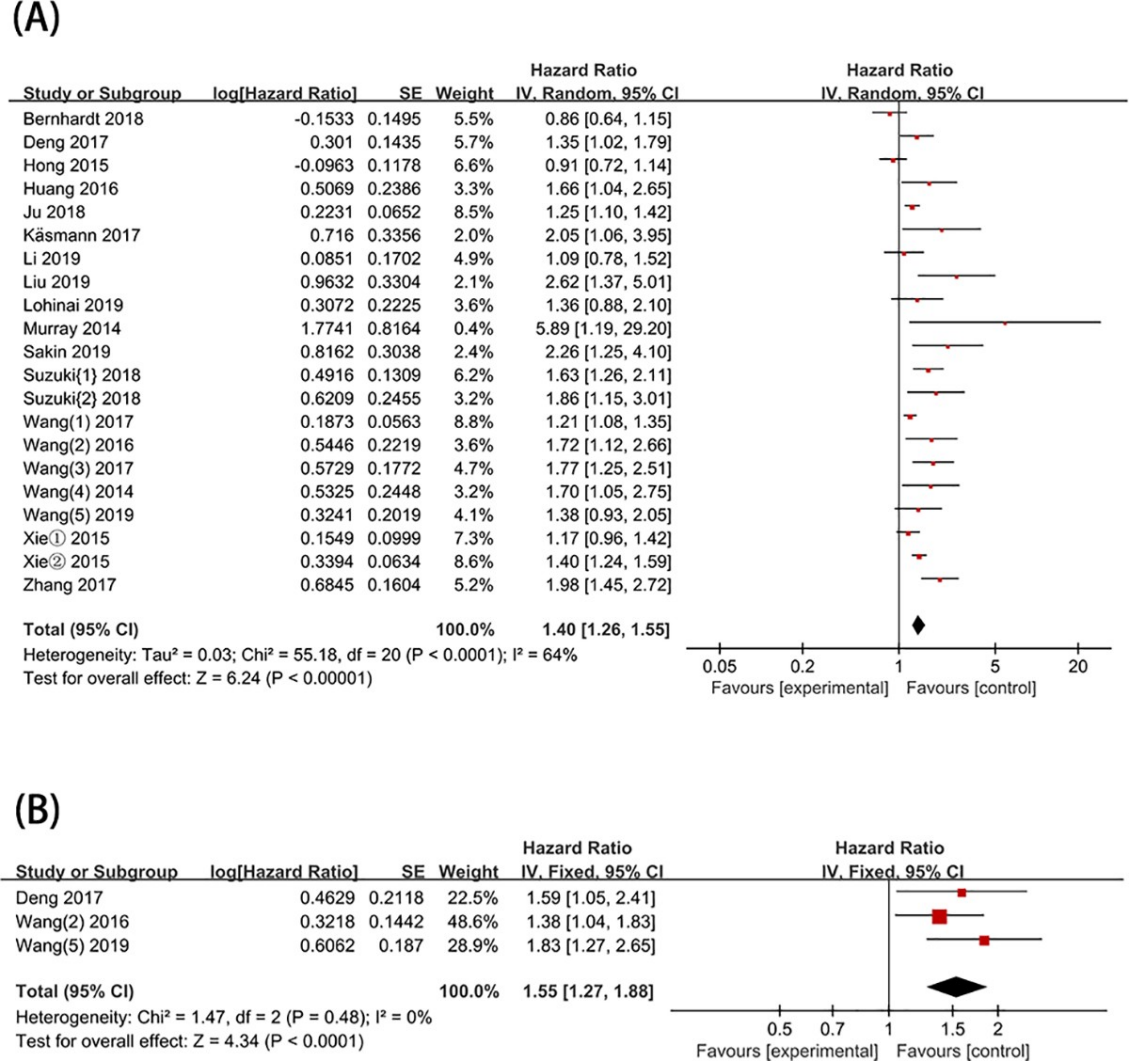
Forest plot of HR for the association of pretreatment NLR in patients with SCLC. (A) OS; (B) PFS.

According to the included literature, 20 studies provided data on OS related to pretreatment NLR in patients with SCLC. As shown in Fig 2, a high pretreatment NLR was closely related to poor OS (HR = 1.40, 95% CI = 1.26–1.55, *P* < 0.00001; I^2^ = 64%; Fig 2). In the heterogeneity analysis, significant heterogeneity was observed among the included studies. Therefore, subgroup and meta-regression analyses were required to explore the source of heterogeneity.

Data on PFS related to pretreatment NLR in patients with SCLC were provided by 3 studies. The pooled result showed that high pretreatment NLR was closely related to PFS (HR = 1.55, 95% CI = 1.27–1.88, *P* < 0.0001; I^2^ = 0%) (Fig 2).

### The pretreatment NLR and clinicopathological features of SCLC

As shown in Table 2, the pretreatment NLR was significantly associated with clinical stage of SCLC (OR = 2.14, 95% CI = 1.35–3.39, *P* = 0.001). However, significant association between the pretreatment NLR and SCLC was not found in sex, age, and smoking.

**Table 2:** The association between NLR and clinicopathological features of SCLC.

Variables	Studies	OR [95% CI]	*P* value	Heterogeneity		Model
				I^2^ (%)	*P*_2_ value	
(1) Sex (Male vs. Female)	9	0.75 [0.50–1.15]	0.19	63	0.006	random
(2) Age (≥ 60 vs. < 60)	4	0.81 [0.38–1.73]	0.58	80	0.002	random
(3) Clinical stage (E vs. L)	7	2.14 [1.35–3.39]	0.001	67	0.005	random
(4) Smoking (Yes vs. No)	6	0.98 [0.63–1.51]	0.92	53	0.06	random

L:limited stage; E:extensive stage

### Subgroup and meta-regression analyses

To elucidate the source of heterogeneity between studies, we performed subgroup analyses by clinical stage, ethnicity, cut-off values for NLR, and sample size (Table 3). Through subgroup analysis, the prognostic role of pretreated NLR in OS did not change significantly (Table 3), and significant heterogeneity remained between most subgroups. We used meta-regression analysis for quantitative analysis. The results of the univariate analysis revealed that sample size (*p*_3_ = 0.017) partly explained the source of heterogeneity (Table 3). Multivariate analysis (*p*_3_ = 0.031) also showed that the sample size may be the main source of heterogeneity (Table 3).

**Table 3:** Subgroup and meta-regression analyses between NLR and OS.

Variables	Number of outcomes	HR [95% CI]	*P* value	Heterogeneity		Model	*P*_3_-value of Meta-regression	
				I^2^ (%)	*P*_2_ value		Univariate	Multivariate
(1) Ethnicity							0.329	0.762
Asian	14	1.35 [1.22–1.51]	< 0.00001	61	0.001	random		
Caucasian	7	1.40 [1.16–2.21]	0.005	71	0.002	random		
(2) Cut-off value							0.659	0.476
< 4.0	12	1.30 [1.21–1.39]	< 0.00001	40	0.08	fixed		
≥ 4.0	9	1.36 [1.11–1.67]	0.003	78	< 0.0001	random		
(3) Sample size							0.017	0.031
N < 150	6	1.89 [1.50–2.38]	< 0.00001	0	0.73	fixed		
150 < N ≤ 200	6	1.26 [1.16–1.36]	< 0.00001	30	0.21	fixed		
N > 200	9	1.34 [1.11–1.59]	0.002	77	< 0.0001	random		
(4) Clinical stage							0.563	0.925
L	5	1.47 [1.03–2.10]	0.04	75	0.003	random		
E	3	1.51 [1.08–2.10]	0.02	72	0.03	random		
L + E	13	1.38 [1.22–1.57]	< 0.00001	61	0.002	random		

L:limited stage; E:extensive stage

### Sensitivity analysis

**Fig 3:**
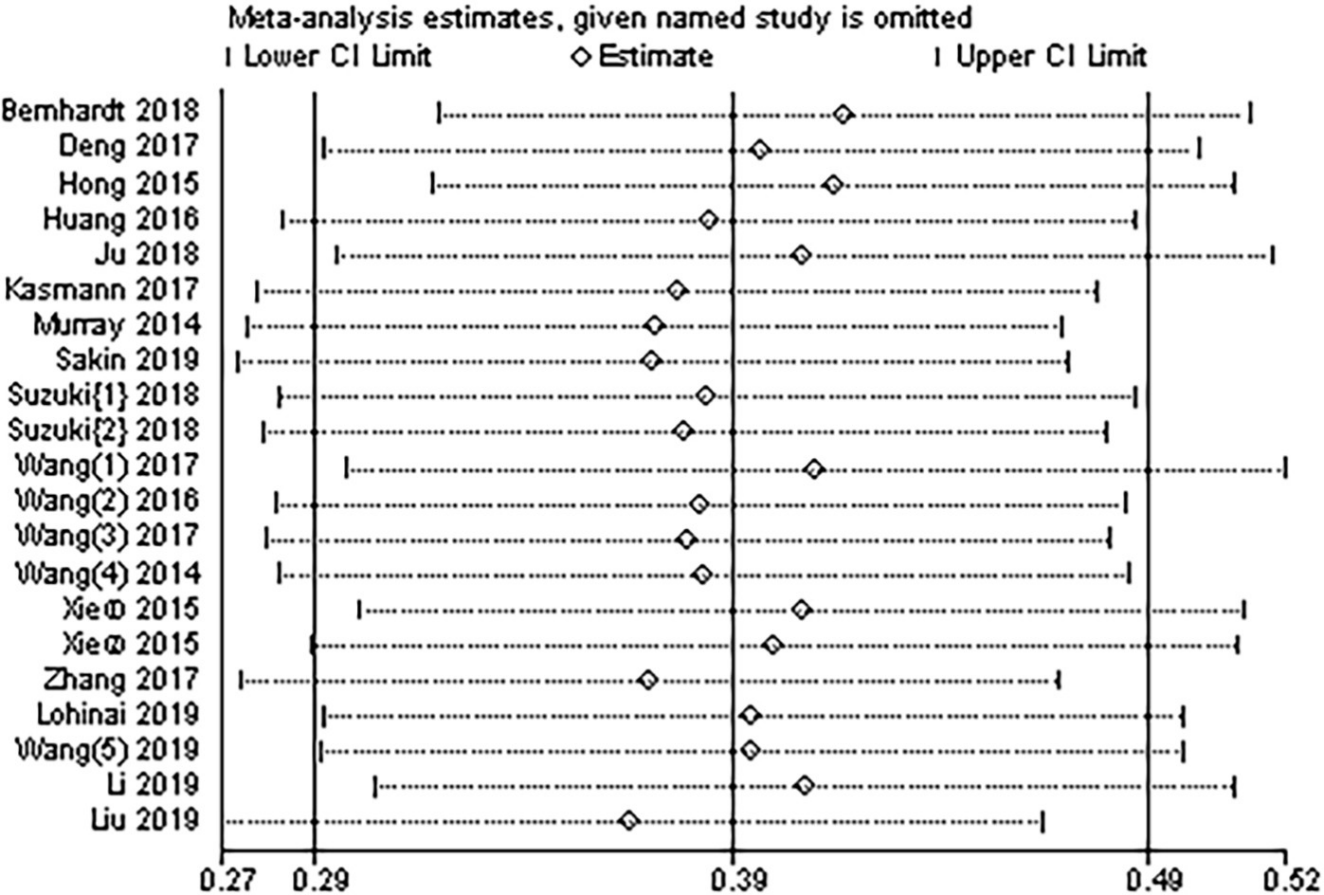
Sensitivity analysis of the relationship between pretreatment NLR and OS in patients with SCLC.

We conducted a sensitivity analysis to assess whether individual studies affected the overall analysis. Our results showed that any of the studies could be removed, and the remaining HRs of the combined studies remained within the 95% CI of the combined HR in the meta-analysis (Fig 3). These findings indicated that the meta-analysis had good stability.

### Publication bias and the trim and fill method

**Fig 4:**
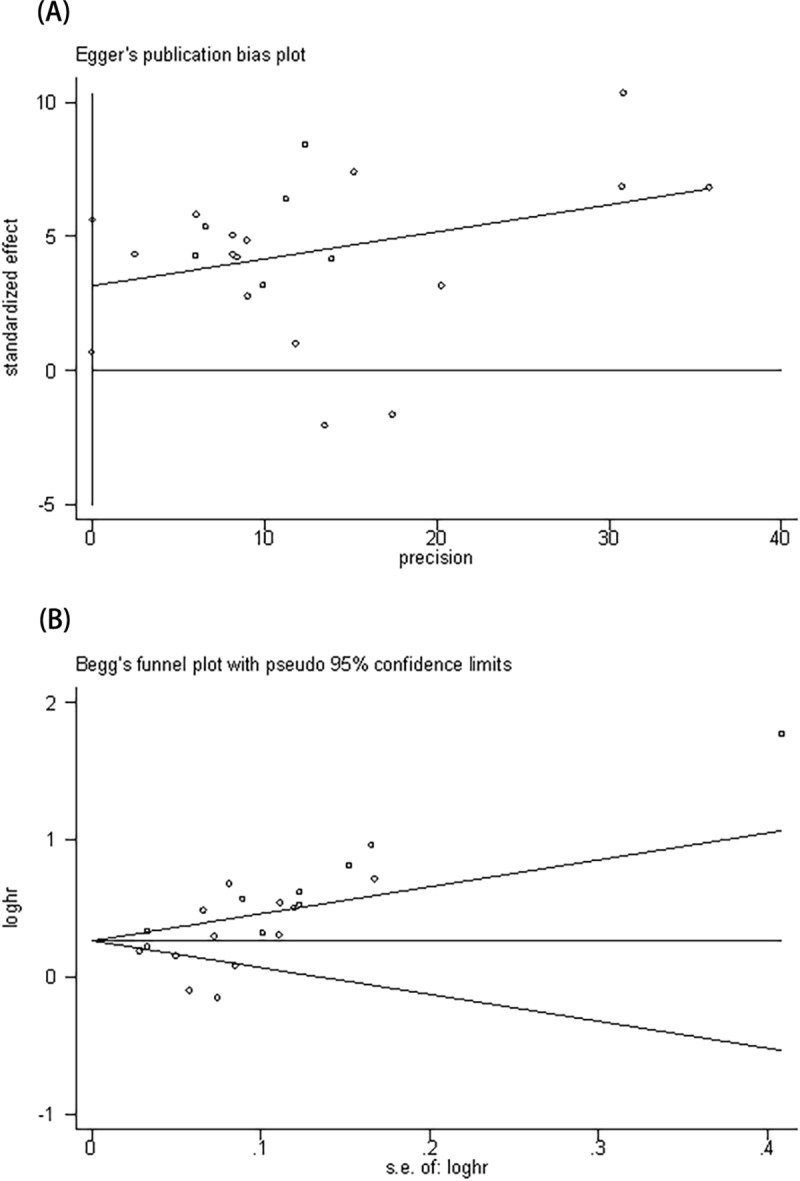
Funnel plot for analysis of publication bias. (A) Funnel plot developed using the Egger method; (B) funnel plot using the Begg method.

**Fig 5:**
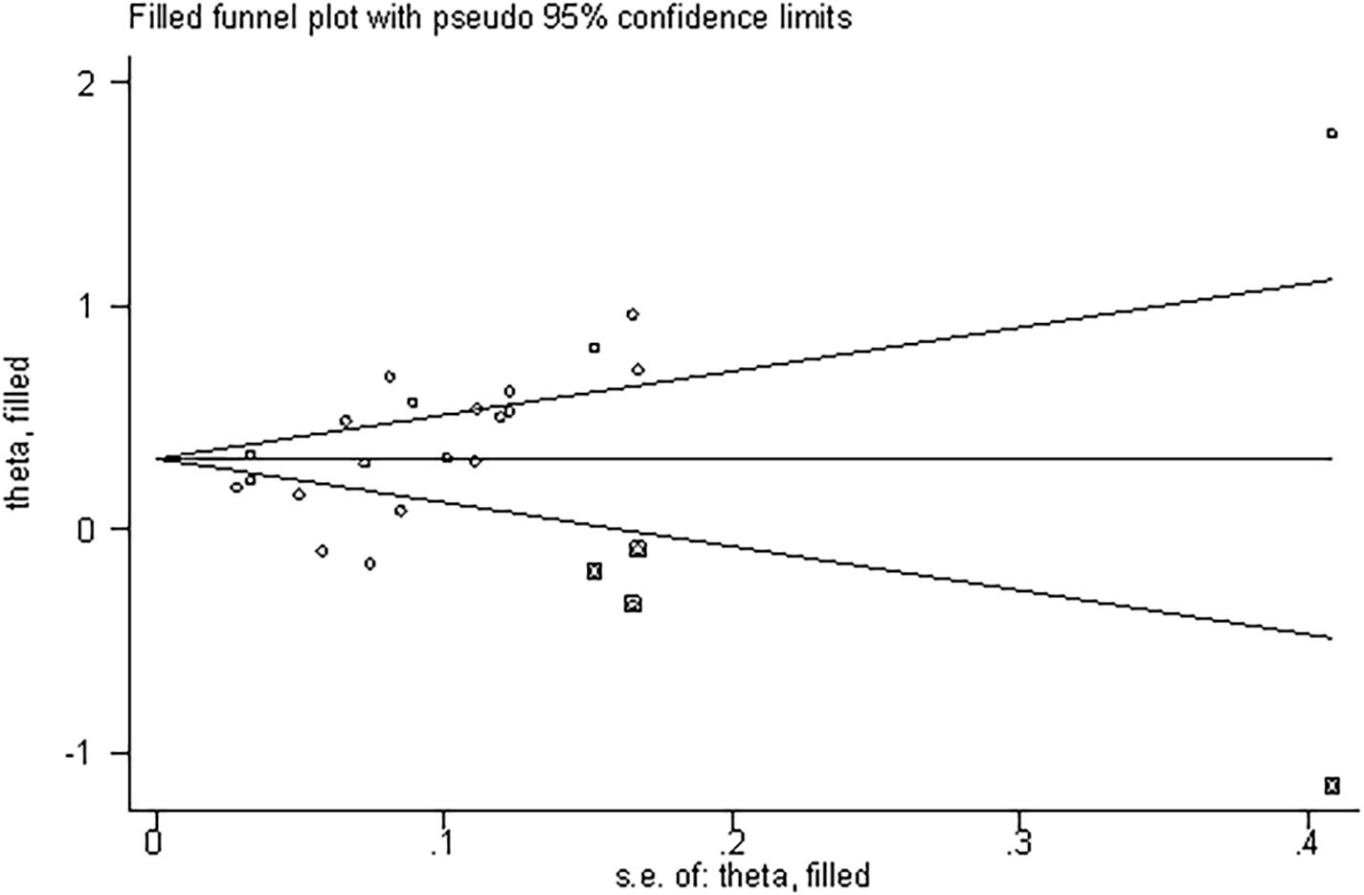
Funnel plot adjusted by the trim and fill method.

Quantitative analysis of publication bias was performed using the Begg method and the Egger method. The results showed the presence of a significant publication bias (Begg’s test: *P* = 0.015; Egger’s test: *P* = 0.015; Fig 4). Therefore, we used the trim and fill method to estimate the asymmetry in the funnel plot. After filling four unpublished studies by calculation, the funnel plot was symmetrical (S1 Text). No statistically significant change was observed in the results (Fig 5).

## Discussion

The tumor-associated inflammatory response is a potential prognostic indicator prior to treatment, manifesting as peripheral blood neutrophilia and relative lymphopenia [40]. Pretreatment NLR is readily available to assess the prognosis of patients with SCLC without high costs, allowing patients and physicians to make informed decisions before clinical treatment.

By pathological typing, lung cancer can be divided into small cell lung cancer and non-small cell cancer. Small cell lung cancer is a separate category, which has special biological behavior and clinical characteristics. Previous meta-analyses[41, 42] have focused on the relationships between NLR and prognosis in patients with non-small cell lung cancer. Additionally, the prior meta-analyses of SCLC [43, 44] only included two studies, which have not explored the clinicopathological value of NLR in SCLC. In our current meta-analysis, the conclusions presented herein identified 16 studies with 17 outcomes, most of which were published in the last two years and showed pretreatment NLR could provide a clinical reference for predicting prognosis in patients with SCLC.

The meta-analysis assessed the clinicopathological and prognostic value of the pretreatment NLR in SCLC. The results suggested that pretreatment NLR levels were inversely proportional to PFS and OS (PFS, HR = 1.55, 95% CI = 1.27–1.88, *P* < 0.0001; I^2^ = 0%; OS, HR = 1.40, 95% CI = 1.26–1.55, *P* < 0.00001; I^2^ = 64%). However, although the pooled result of PFS with only includes two studies has no apparent heterogeneity, the result requires larger sample studies to validate. At the same time, the Pooling analysis found a significant association between pretreatment NLR and clinical stage. High pretreatment NLR is a risk factor for extensive-stage small cell lung cancer.

However, this meta-analysis also had some limitations. First, significant heterogeneity was observed. The main source of heterogeneity by meta-regression analysis was the sample size. Subgroup and sensitivity analyses were used to confirm that the results of this meta-analysis were stable. Second, the papers showed publication bias, and affirmative results are easier to publish. Importantly, however, after correction with the trim and fill method, there were no changes in the prognostic value of pretreatment NLR in patients with SCLC. Finally, the clinical data between pretreatment NLR and PFS with SCLC is relatively small. Therefore, more large-scale prospective studies are needed.

## Conclusion

The meta-analysis showed that high pretreatment NLR was a risk factor for extensive-stage small cell lung cancer. Moreover, high levels of pretreatment NLR were significantly associated with reduced PFS and OS in patients with SCLC and that NLR was readily available and less costly, suggesting that NLR could be used as a biomarker for the prognosis of SCLC.

## Supporting information

S1 Checklist PRISMA checklist. (DOC)

S1 Table Search strategy of PubMed. (DOCX)

S1 Text The process of the trim and fill method. (DOCX)
