# Supplementary material for: The clinicopathological and prognostic value of the pretreatment neutrophil-to-lymphocyte ratio in small cell lung cancer: A meta-analysis
Source: PLoS One. 2020 Apr 2;15(4):e0230979. doi: 10.1371/journal.pone.0230979 (PMC7117946; doi:10.1371/journal.pone.0230979)
Supplement: S1 Table — (DOCX) [file pone.0230979.s002.docx]

| **Database** |  | **Search terms** |
| --- | --- | --- |
| **PUBMED** |  | **Neutrophil-lymphocyte ratio** |
|  | 1 | Neutrophil-lymphocyte ratio[Title/Abstract] |
|  | 2 | NLR[Title/Abstract]) |
|  | 3 | Neutrophil lymphocyte ratio[Title/Abstract] |
|  | 4 | Neutrophil to lymphocyte ratio[Title/Abstract] |
|  | 5 | or/1-4 |
|  |  | **Small Cell Lung Carcinoma** |
|  | 6 | Small Cell Lung Carcinoma [Mesh] |
|  | 7 | Lung Cancer[Title/Abstract] |
|  | 8 | Lung Carcinoma[Title/Abstract] |
|  | 9 | Small Cell Lung Cancer[Title/Abstract] |
|  | 10 | Oat Cell Lung Cancer[Title/Abstract] |
|  | 11 | Small Cell Cancer Of The Lung[Title/Abstract] |
|  | 12 | Carcinoma, Small Cell Lung[Title/Abstract] |
|  | 13 | Oat Cell Carcinoma of Lung[Title/Abstract] |
|  | 14 | or/6-13 |
|  |  | **Combination** |
|  | 15 | 5 and 14 |
