# Supplementary material for: The clinicopathological and prognostic value of the pretreatment neutrophil-to-lymphocyte ratio in small cell lung cancer: A meta-analysis
Source: PLoS One. 2020 Apr 2;15(4):e0230979. doi: 10.1371/journal.pone.0230979 (PMC7117946; doi:10.1371/journal.pone.0230979)
Supplement: S1 Text — (DOCX) [file pone.0230979.s003.docx]

**Meta-analysis**

| Method | Pooled | 95% CI | | Asymptotic | | No. of |
| --- | --- | --- | --- | --- | --- | --- |
|  | Est | Lower | Upper | z_value | p_value | studies |
| Fixed | 0.266 | 0.239 | 0.292 | 19.704 | 0.000 | 21 |
| Random | 0.393 | 0.295 | 0.491 | 7.854 | 0.000 |  |

Test for heterogeneity: Q= 219.320 on 20 degrees of freedom (p= 0.000)

Moment-based estimate of between studies variance = 0.042

**Trimming estimator: Linear**

**Meta-analysis type: Random-effects model**

| iteration | estimate | Tn | # to trim | diff |
| --- | --- | --- | --- | --- |
| 1 | 0.393 | 136 | 2 | 231 |
| 2 | 0.351 | 148 | 3 | 24 |
| 3 | 0.331 | 155 | 4 | 14 |
| 4 | 0.316 | 161 | 4 | 12 |
| 5 | 0.316 | 161 | 4 | 0 |

**Filled**

**Meta-analysis (exponential form)**

| Method | Pooled | 95% CI | | Asymptotic | | No. of |
| --- | --- | --- | --- | --- | --- | --- |
|  | Est | Lower | Upper | z_value | p_value | studies |
| Fixed | 1.290 | 1.257 | 1.324 | 19.089 | 0.000 | 25 |
| Random | 1.376 | 1.248 | 1.516 | 6.415 | 0.000 |  |

Test for heterogeneity: Q= 256.721 on 24 degrees of freedom (p= 0.000)

Moment-based estimate of between studies variance = 0.047
